# The Yellow Fever

**Published:** 1803-02-01

**Authors:** 


### THE YELLOW FEVER.

We have been favoured by Dr. PATTERSON, of Londonderry with the following interesting correspondence which has taken place between him and Dr. MILLER, Editor of the Medical Repository of New York. The respectability of the parties in this controversy and the importance of its subject will recommend their arguments to the perusal of our readers,

Dr. PATTERSON *having enclosed some printed letters to* Dr. MILLER *for publication in the New York Repository, that gentleman inserted the following observations on those letters in his Repository.*

( *New York Medical Repository, vol. iv. p.* 83. )

" Dr. William Patterson, of Londonderry, in a letter to Dr. Miller, has been so obliging as to enclose a printed copy of two letters, one from Dr. William Drennan, of Dublin, the other from himself, addressed to Joseph Wilson, Esq. American Consul, on the subject of Yellow Fever. We are sorry our limits do not allow us to re-publish these letters in the present number. These estimable gentlemen certainly manifest the most benevolent wishes for the deliverance of America from pestilential diseases, and a warm and active spirit of philanthropy. But they do not seem to have taken the requisite pains to obtain information on a subject which calls forth all the fervour of their zeal, and all the tenderness of their commiseration.

" The opinions of Dr. Drennan are evidently predicated on the *specific and permanent contagiousness*, and those of Dr. Patterson on the *importation* of yellow fever. These opinions reciprocally depend upon each other, and, of consequence, must stand or fall together. This is neither the proper time nor place to enter fully into such a discussion. If medical gentlemen are determined to consider a disease as specifically contagious, merely because it affects many people at the same time, they may find examples of such contagion in the intermittents of a marsh, in the pneumonia and phthisis of winter, or in the gastritis of a convivial circle, whose viands are converted into poison by the admixture of arsenic. It gives us pain to see such respectable physicians precipitately rushing into conclusions altogether unwarranted by the premises. If they read the latest and best performances of British physicians on the diseases of the West Indies, they will find that the yellow fever is not a contagious distemper. Every merchant and planter in those islands knows this fact. We assert that it is as little contagious in the United States. It only prevails within certain local ranges of air contaminated by the effluvia of animal and vegetable putrefaction; and, beyond these limits, is incapable of propagation.* Multitudes take the disease in our cities, who never approach the sick, the suspected vessels, or any fomites supposed to be imbued with contagion. The only possible mode of accounting for these cases, is to ascribe the illness to the patient's immersion in an atmosphere greatly vitiated throughout certain definite portions. Our present number (for May, June, and July, 1800,) affords several authentic instances of the same pestilential distemper in the interior of our country, where importation was impossible, and where local circumstances completely solve the difficulty. Dr. Drennan's plan of extinguishing the disease by high artificial heat would not succeed unless applied to a whole city, which he will grant to be impracticable; and Dr. Patterson's doctrine of importation is insufficient to explain the ravages of yellow fever at Galliopolis, and at Bald Eagle Valley, in Pennsylvania, and at many other places.

"These gentlemen seem to be surprised that so little has been attempted by public authority, and so little accomplished by the medical profession in the United States. But to infer imbecility in the government, or incapacity among physicians, because the havock of yellow fever has not been arrested, is, in our judgment, to take a narrow view of the subject. The devastation of pestilence forms a mournful page in the history of every age and nation. It is to be lamented that American skill should be in any degree

---

* "In asserting that the yellow fever is not contagious, let us be explicitly understood. We do not deny that the excretions of the sick, in this disease, where cleanliness is not duly observed, may assist, like any other kind of animal putridity, in the formation of pestilential matter. But we contend that this is not more likely to happen from the effluvia of a patient in yellow fever, than from the effluvia of one labouring under any other fever, or from the putrid vapours emitted by a gangrenous ulcer, or by a heap of dead and putrefying animal and vegetable substances, under similar conditions of a hot, moist, and otherwise insalubrious atmosphere. How far this differs from the constitution of specific contagion, such as that of small-pox and measles, every reader will be able to judge."

degree baffled; but surely it is not without accumulated precedents. The energies of government, aided by all the wisdom of the illustrious Sydenham and Morton, were insufficient to stay the destruction of the plague in London, in 1665. All the learning, diligence and zeal of the physicians of His Britannic Majesty's forces in the West Indies, have not prevented those islands from continuing to be the grave of Europeans. And, if we are not misinformed, the mortality of scarlatina anginosa, in some parts of England, has lately extended to nearly one-fourth of those who were attacked, notwithstanding all the splendid improvements of medical science in Great Britain.

"While we thus freely animadvert upon the opinions of these gentlemen, we cannot forbear to repeat our high respect for their talents, as well as their motives on this occasion; and we shall be extremely happy to receive their further communications on the subject."

---

Dr. PATTERSON *feeling himself called upon to animadvert on these Observations by* Dr. MILLER, *has since addressed the following Paper to the Editors of the Medical Repository, at New York, in which work it has not yet appeared.*

READING in the Fourth Volume of the Medical Repository, p. 83—85, some Strictures on two printed letters, relating to Yellow Fever, transmitted from Londonderry; Dr. Patterson, the writer of one of those letters, feels himself particularly concerned in noticing the construction which the Editors of that respectable Work have put on the humble hint, which he addressed to the American government through the medium of their Consul in this country. He takes leave to assure them, that he did not entertain the smallest idea of imputing 'imbecility' to that government, either in regard to inattention concerning yellow fever, or concerning any other object belonging to their administration. On the contrary, he is strongly prepossessed in favour of both the wisdom and energy of the public councils and sovereignty of the American States. He conceives that he does not transgress against the respect and deference due to, and which he will always maintain for, the constituted authority of every social union, by his concurring in the recommendation of a measure which consults the welfare of a rising and meritorious people, and to which, as far as he could observe, the legislative power of that people had not effectively adverted.

In

In this case, Dr. P. feels himself actuated by the principles of the British constitution, which he is warranted in considering superior to many, and inferior to none, in the civilized world, for wise polity and rational freedom. Agreeably to the principles of this constitution, it is the undoubted right, and, in certain circumstances, like that now before us, it becomes the duty of every intelligent subject, to point out to the servants of the crown, by memorial or otherwise, such plans and measures as have a tendency to benefit the nation at large; observing, however, in the exercise of this right, a modesty proportioned to the distance between the persons addressed and the person addressing. These principles of government, and rules of decorum, accord with the law of nations and the spirit of philanthropy, which sanction the member of a foreign state, who does not trespass against them, in offering some of the fruits of his researches to his fellow-men in any region. That Dr. P. has not been guilty of such a trespass, he expects every candid reader, after perusing his letter to the American Consul, will do him the justice to admit; and he entertains too high an opinion of the wisdom and liberality of the American executive, to suppose that it would, in a question of health, deride, or take amiss, the suggestions of a dispassionate and well-intentioned observer, living in any country, who has no selfish systems to establish, nor private interests to serve.

Dr. P. now turns to his professional brethren, the medical faculty in America, and protests that, in assigning the causes which operated against the settlement of opinion amongst them, he did not mean to detract from their merits, either as men or as physicians, for in both capacities he holds them in considerable estimation; nor does he see how his expressions can fairly be interpreted in a sense contrary to this sentiment of regard. Difference of opinion, and a desire to examine a disputed point in science, are not evidences of a contentious temper; nor do they imply a charge of 'incapacity' against an opponent. Men earnest in the pursuit of truth and knowledge, do not argue merely for argument's sake; they do not prefer railing to reasoning, nor do they court discussion with persons of 'incapacity,' from whom they cannot derive either information or credit.

As no man is exempt from error, nor any set of men infallible, the Editors consequently ought to allow, that Dr. P. may *differ* from them and yet *not* be in the *wrong*; and *agree* with them and yet *not* be in the *right*, especially in an intricate and unsettled point; for whilst there are men, there will be difference of opinion; and as long as as the difference subsists, he must be a very wise man indeed, who is certain that all mankind are wrong except himself. Medicine in general, and the case under consideration in particular, afford striking illustrations of this reasoning. Does dogmatic confidence in our own opinions ever stand upon more slippery ground? or, is it more exposed to ridicule, than when those opinions concern the art of medicine, or relate to a medical question at once complicated and obscure? That the nature and propagation of morbid effluvia, especially of those exciting febrile action in the human body, are yet involved in doubt and difficulty, the early, successive, and recent controversies on the subject sufficiently demonstrate. But, at this time of day, physicians are not like Casuists, amongst whom, the support of one grave Doctor is enough to stamp an opinion with almost unlimited currency.

Though Dr. P. has not been an eye-witness of the phenomena constituting Yellow Fever, and its train of consequences, he is not unacquainted with the opinions of men of high medical authority, who have been eye-witnesses; and who maintain the *doctrine of contagion*, or of a peculiar morbid effluvium, germinated in the human body, being the essential cause of that distemper. The facts stated by these observers he compares with the facts stated by observers of an opposite persuasion, and, with the occasional assistance of analogy, he draws conclusions upon which he founds his opinion. Thus, experience corrects analogy, analogy amends experience, and reasoning tries both. This appears to be as rational a method as any, for arriving at truth in almost every species of human research, and signally so in medical cases, of a subtile character, such as that now at issue. By this method, the delusions arising from what is called *false experience* are avoided; a chain of substantial postulates is obtained; and a valid induction is formed, which shapes, trains, and ratifies a doctrine.

Following this rule of inquiry, the replyer has embraced an opinion of the contagious nature of that pestilential distemper, denominated Yellow Fever; an opinion on which, from the state of information in which he at present stands, he is *not yet* disposed to retract. He does not suppose with the vulgar, that an infectious fever is a disease which seizes every person, *indiscriminately*, who comes within its sphere of action; the plague itself does not possess this sweeping influence. Nor does he confine his ideas of contagion to an 'emanation *directly* arising from the body of a man under a particular disease, and exciting a similar a similar disease in the body of the person to whom it is applied.' But, with Dr. Lind, he would define an infectious fever, 'A distemper, which, in certain circumstances, is, or may be, communicated to one, two, or more persons, and that generally in its identical form and peculiar nature.' Facts and observations, which, in his mind, are convincing, render this definition applicable to Yellow Fever. This disease has been repeatedly traced to seats and matters, which historical records, and recent experience, unite in deeming the fountains, reservoirs, or conduits of contagious effluvia.

Early in the last century (the 18th) the Yellow Fever prevailed in Charlestown four times in twenty-five years, and every time it was traced to some person who had lately arrived from the West-India islands, where it was epidemical. In 1760, during the French war, it first fully unveiled itself in Philadelphia, and was traced to arise from the clothes of a young man who died of it in Jamaica, which were sent to his friends in that city; those friends were the first who fell victims to it, and though it spread to others, its ravages were not very extensive. Afterward it was not known in America till the year 1793, when its introduction was shewn to have originated from several vessels which were at Cape Francois, during the dreadful capture of that place by the Negroes, and which vessels conveyed a number of the wretched inhabitants, with their goods of every description, to Philadelphia. In 1794, it appeared at Baltimore, where it was brought by a ship from the West Indies; and at New York, in 1795, when it was also found to have issued from a West India vessel. In 1797, it again made its appearance in Philadelphia, and was clearly followed up to a vessel from St. Domingo. During the year 1798, it visited not only Philadelphia and New York, but many other of the commercial towns, in all of which it has been traced to the cause now mentioned; and consequently, it is to be presumed, that its visitation of Norfolk, Baltimore, Charlestown, and Providence, in the present year, is deducible from a similar descent. Nay, its extensive effects in the United States of America, might with reason be adduced as one proof of its comparative novelty, and of its origin from a foreign cause.

The Editors "do not deny that the excretions of the sick, in this disease, where cleanliness is not duly observed, may assist, like any other kind of animal putridity, in the formation of pestilential matter. But we contend (say they) that this is not more likely to happen from the effluvia fluvia of a patient in yellow fever, than from the effluvia of one labouring under any other fever, or from the putrid vapours emitted by a gangrenous ulcer, or by a heap of dead and putrefying animal and vegetable substances, under similar conditions of a hot, moist, and otherwise insalubrious atmosphere." Here the Editors at once admit and maintain, that two different causes are productive of one and the same effect; morbid excretions, animal putridity, and vegetable putrefaction, under certain unwholesome states of the atmosphere, may, it seems, generate *pestilential matter*; thus saying, that a *something else* than the common corruptions of the air, by putrefactive processes, is necessary to constitute febrile contagiou. That a something else is requisite for this purpose is certain; for febrile diseases, every one knows, are communicated and spread by emanations from the sick, whose excretions have not the smallest sensible putrid odour or qualities; whilst, on the other hand, putrefaction may be present, in a very high degree, without begetting contagion, as may be daily observed in sundry common manufactories, &c.

Though Dr. P. has not read all the latest and best performances of the British physicians on the diseases of the West Indies, he has met with authorities from that quarter, which convince him that Yellow Fever is there of an infectious nature. Dr. Warren, who resided several years at Barbadoes, observed, so early as the year 1723, that it was a foreign intruder there; that it could not be ascribed to any vitiation of the air from lakes, marshes, or woods, because the land was even then well cultivated, and entirely free from lakes and marshes, without a sufficiency of wood in fact for fuel; and that the disease was propagated by infection, notwithstanding the air of the island was remarkably fresh and pure. Dr. Chisholm, an intelligent and experienced practitioner, and surgeon to the British ordnance in Grenada, traces its route from Boullam on the coast of Guinea, by Philadelphia and New London, into the West Indian islands, where its infectious character became indisputable, and its temper malignant.

There is reason to believe, that mistakes, relative to the real nature of Yellow Fever, have arisen from confounding it with the violent remittents of the West Indies and America, as the former is infectious both in those islands and in that continent, and the latter are not so. Why, it may be asked, did the Americans, from the ingress of the fever, in 1793, to the present day, uniformly guard, with the utmost vigilance and rigour, all the avenues, leading from from their towns infested with it, to those at the same time free from it? The reason is plain.—Because the magistracy and the great body of the people were convinced by experience and observation, that the malady is procreated and spread by a specific contagious matter.

If Yellow Fever be the offspring of street filth, spoiled beef, rotten coffee, or other substances in a similar state of depravity, why does it appear where these causes are not to be found, and keep off where they predominate? Why did it break out in Kensington, Chester, and Wilmington, which enjoy all the advantages of country air, and complete ventilation? In former times, when the police was not so good as it now is, and during the American contest, when the streets of Philadelphia were often covered with filth of every kind, if the distemper be a native one, why did it not then shew its face? If it be engendered by those contaminations of the air, why did the flats of Baltimore, or the fens of Charles-Town, or Savanna, escape, whilst Boston, New London, and Wilmington, were sinking under its overwhelming power? If the sensible qualities of the air, as heat, cold, moisture, or dryness, were parents of the distemper, why has it sprung up in seasons and periods widely differing from one another in these respects? In 1795, during the hot season at New York, the weather was warm and moist, but there was very little rain; in the hot season of 1798, at the same place, the weather was not only much warmer, but there were heavy rains with considerable thunder; yet, in both these years, the fever reigned in that city.—A city too, which, with the beauties of Nature, combines a healthful situation, salutary municipal laws, and a supply of choice water.

The pestilential fever *generally* breaks loose, in America, in the hot summer months, and is repressed by autumnal cold; yet, from the testimony of Dr. Rush, it appears that it commenced, in Philadelphia, in the cold weather of November, 1794. Surely, then, it is rather paradoxical to say, that a disease, which starts up in both hot and cold weather, or not only in July but in November, is germinated and fostered by the operation of two such opposite causes. Can any thing be more like the agency of a peculiar febrile effluvium, than this property of appearing in contrarious conditions of the air depending on the impressive circumstances of temperature? The answer may be given from the analogy of variolous contagion, which sets out in all weathers, and seasons of the year. As to the appearance of a few sporadic cases of the disease, in the current year year (1800), in New York, (in spite, it seems, of strict *quarantine* injunctions,) Dr. P. has not yet heard enough of particulars to form a judgment on the occasion. But were he to hazard any opinion, in this instance, he would conjecture that all its circumstances may be hereafter explained on the principles of the doctrine which he here defends. He likewise apprehends, that the eruption of the fever, in 1796, at Galliopolis, near the river Ohio, and, in 1800, at Bald Eagle Valley, in Pennsylvania, may be accounted for on the same principles. From what has been already said, the Editors may perceive, that for examples of contagion, recourse has not been had to the intermittents of a marsh, the pneumonia or phthisis of winter, nor the poisonous viands of a festive table.

Adverting now to the sanctions of analogy, it appears that several cities in the south of France and in Italy, especially Marseilles, Milan, Genoa, and Leghorn, abound with such foul appendages as are said to generate pestilential fever in the towns of America, without experiencing that calamity. If improvements have been introduced into these European towns, they are certainly not such as have destroyed the causes usually assigned for creating disease, and are confined to such parts of them as are newly constructed. In the old parts of those towns, the streets are narrow, badly paved, and often not paved at all, whilst they are the general receptacle of every kind of filth, without the attention of a police to cleanse them. It is even a custom to keep them constantly strewed with hay and straw, that by the rain, and the passage of people, horses and carriages, those materials may be converted into manure. Besides, all who have visited these towns must have been struck with the want of common sewers, the height and crouded construction of the houses, the numerous families with which they are filled, the rooms seldom cleansed, and the walls covered with cotton and silk tapestry instead of plaster. Add to this, the aggravating pollutions of several years warfare, to which those places have been more or less subjected. If, then, under such unfavourable circumstances, those European towns, situated in one of the hottest climates, where the winters often are exempt from frost, not only have never known either Yellow Fever, or any disorder of the kind, to originate among them, but have been able entirely to rid themselves of the plague by their quarantines, and continue healthy ever since these regulations were adopted, it affords the strongest arguments against the origin of that fever in the towns of America.

Pools

Pools of stagnant water, mill ponds, and the like, have been accused for giving birth to yellow fever. On the continent of America, in some of the northern counties of the state of New York, for instance, the retreat of the water, which had overflowed considerable tracts of low grounds in the vicinity of mill-dams, left the surface of those grounds exposed to the influence of the sun; the consequence of which was, a violent bilious remitting fever, which attacked numbers within a certain distance of the source; but the mill-dams being destroyed, and the waters suffered to flow in their usual channels, the country regained its former measure of salubrity. It does not appear, however, that the sickness thus produced was at all suspected to be Yellow Fever, as no hint to this purpose is given by the narrator, who, on the contrary, as here noticed, classes it amongst the tribe of bilious remittents; fevers characterised by essential features of distinction from that pestilence. Even in the hot climate of Egypt, whilst the waters of the Nile inundate the land, the people are healthy, and diseases do not arise until the waters subside, leaving a vast extent of surface covered with noxious matter, to be exalted and exhaled by the solar heat; yet, as we are informed by late and intelligent travellers, the plague is not an endemic of that country, but that the peculiar diseases are leprosy, affections of the eyes, &c.

The doctrine maintained by the Editors, though not entirely new, has so much of novelty in it, and is supported by so much ingenuity and abilities, that it has a powerful tendency to subvert settled opinions, and to lead nations to abandon practices, in which they have placed much dependence, and which have been generally employed with success for reducing and subduing contagious matter. It is of the utmost importance, therefore, that a doctrine, the more seductive by being thus ingeniously and ably displayed, should be well founded; which can be accomplished only by means of careful examination and candid discussion, under the discipline of observation, reasoning, and science.

### To the Reader.

In corroboration of the sentiments contained in the foregoing *Reply*, Dr. Patterson begs leave to refer the reader to Vol. vii. of the London Medical Journal, p. 311, 320, where he will find facts and observations of the most applicable description. Dr. P. would also recommend attention to the tract written by Dr. Haygarth, alluded to in the same place, as investigating the *causes* of pestilential epidemics; a knowledge of which is the safest guide for arriving at their prevention and cure.

*Observations*

